# Negligible Toxicokinetic Differences of Glyphosate by Different Vehicles in Rats

**DOI:** 10.3390/toxics11010067

**Published:** 2023-01-11

**Authors:** Yu-Jin Kim, Nitin Nitin, Kyu-Bong Kim

**Affiliations:** 1 College of Pharmacy, Dankook University, 119 Dandae-ro, Cheonan 31116, Chungnam, Republic of Korea; 2 Center for Human Risk Assessment, Dankook University, Cheonan 31116, Chungnam, Republic of Korea; 3 Department of Food Science and Technology, University of California, Davis, CA 95616, USA

**Keywords:** toxicokinetic, glyphosate, polyoxyethylene tallow amine (POEA), tween 20, vehicles

## Abstract

Glyphosate is a non-selective herbicide. Although glyphosate is not acutely toxic, the intake of glyphosate-based herbicides has caused many accidents. Some studies have suggested that surfactants might be the cause. The purpose of this study was to compare the toxicokinetic (TK) properties of glyphosate according to different vehicles in rats. Glyphosate (1%) was dissolved in distilled water (DW), polyoxyethylene tallow amine (POEA), and Tween 20. After a single oral treatment of glyphosate (50 mg/kg), blood was collected at time intervals, and glyphosate concentrations in the target organ (liver and kidney) were determined 24 h after final blood collection. All samples were analyzed using LC-MS/MS. The TK parameters of glyphosate were similar in the DW and Tween 20 groups. However, there were significant differences in T_max_ and volume of distribution (V_d_) between the DW and POEA group (*p* < 0.05). Glyphosate was absorbed about 10 times faster in POEA group rather than DW, and exhibited a higher distribution. However, other important TK parameters of T_1/2_, AUC, and C_max_ were not statistically different among the different vehicle groups. Although glyphosate concentration in the liver was significantly higher in the POEA group than in the DW group, there was no significant difference in the kidney. These results indicate that the toxicokinetics of glyphosate are not significantly affected by POEA. It can be concluded that POEA toxicity itself can be attributed to the acute toxicity of glyphosate-containing products.

## 1. Introduction

Glyphosate (CAS No. 1071-83-6) was first synthesized as a pharmaceutical compound by Martin in 1950 [1]. Glyphosate is a broad-spectrum systemic herbicide and crop desiccant, and is a very simple molecule similar to the amino acid glycine, with one phosphate [2]. Because it can effectively remove all plant types, including creepers, shrubs, and trees, it is used not only in agriculture but also in household gardening [2]. Glyphosate has a molecular weight of 169.07 g/mol and a log Pow of −3.4, making it highly soluble in water [3]. Detailed physicochemical properties of glyphosate and its major metabolite, AMPA are given in Table 1. Glyphosate interferes with the synthesis of phenylalanine and tyrosine in plants. These amino acids are synthesized through a special route called the shikimic acid pathway. Since 5-enolpyruvylshikimate-3-phosphate synthase (EPSPS), the target enzyme of glyphosate, exists only in plants and microorganisms, inhibition of the shikimic acid pathway by glyphosate occurs only in these organisms [4]. Glyphosate is rapidly excreted by animals and humans, and aminomethylphosphonic acid (AMPA) is a metabolite of glyphosate in plants and animals. AMPA also has a small molecular weight of 111.04 g/mol. a log Pow of −4.7, and is soluble in water [5]. Detailed physicochemical properties of AMPA are also shown in Table 1 [2,6,7]. 

In an acute toxicity study of glyphosate, the median oral lethal dose (LD_50_) of glyphosate in rats was >5000 mg/kg [8]. The European Chemical Agency (ECHA) concluded that it was unacceptable to classify glyphosate as a cause of acute oral toxicity following the reported studies. In another study, F344 rats were administered glyphosate at concentrations of 0, 3125, 6250, 12,500, 25,000 and 50,000 ppm for 13 weeks [9]. In male rats, body weight decreased by 6% and 18% at 25,000 and 50,000 ppm, respectively, and in females by approximately 5% at higher doses. Males showed progressive increases in one or more red cell parameters at concentrations above 12,500 ppm. Serum alkaline phosphatase and alanine aminotransferase levels were elevated to 6250 ppm in males and 12,500 ppm in females [9].

Many accidents are caused by ingestion of glyphosate-based herbicides. However, the glyphosate raw material itself has not been reported to show acute toxicity. Some studies suggest that surfactants used in herbicides may be a major cause [10,11,12]. The effectiveness of these pesticides can be enhanced using surfactants. Therefore, the choice of surfactants or adjuvants is important for pesticides. The surfactant used in the well-known herbicide Roundup**^®^** is a polyethoxylated alkyl amine (POEA) [13]. POEA is widely used in pesticides, drugs and other compounds [10]. The oral LD_50_ value of POEA in rats is 1200 mg/kg, indicating that it is more toxic than glyphosate (> 5000 mg/kg) [14]. Toxicokinetics (TK) is a study performed as an essential test when conducting nonclinical toxicity studies to evaluate internal systemic exposure of chemicals [15]. The major objective of a toxicokinetic test is to study the correlation between the systemic exposure to a test substance during the toxicity study and the dose level and time course in the toxicity study. Another objective is to appropriately evaluate the safety in clinical use by identifying the correlation between exposure data obtained from toxicity studies and toxicological findings [15].

A TK study of glyphosate has been previously reported [16]. However, there have been no studies comparing the toxicokinetics of glyphosate according to different vehicles. The objective of the present study was to elucidate the toxicokinetic properties of glyphosate depending on the vehicles used for the different toxicities between glyphosate and its final product in rats. This is because vehicles not only improve selectivity, efficacy and/or safety during drug delivery, but also affect acute toxicity [17,18,19].

## 2. Materials and Methods

### 2.1. Chemicals

Glyphosate, AMPA and Tween 20 were purchased from Sigma-Aldrich (St. Louis, MO, USA). Polyoxyethylene tallow amine (POEA) was purchased from Fisher Scientific International, Inc. (Waltham, MA, USA). Distilled water (DW), acetonitrile (ACN) and dichloromethane were purchased from Honeywell Burdick and Jackson Co. (St. Harvey, MI, USA). The chemical 4-hydroxyacetanilide was purchased from Tokyo Chemical Industry Co. (Tokyo, Japan) and used as an internal standard (IS).

### 2.2. Vehicles

Selected vehicles were: DW (non-surfactant) and POEA (surfactant used in herbicide products) and Tween 20 (surfactant).

Because of its stability and relative non-toxicity, Tween 20 is used as a detergent and emulsifier in many scientific and pharmacological applications [20]. Tween 20 has low toxicity in rats, with an oral LD_50_ of 37,000 mg/kg [21]. It is approximately 30 times less toxic than the POEA used in the product.

Before proceeding with the experiment, the degree of dissolution of glyphosate was determined. Consequently, the completely dissolved state was found to have a 1% concentration of glyphosate. Using the same glyphosate concentration for comparison with other vehicles, glyphosate (1%) was dissolved in DW, POEA (15%), and Tween 20 (15%). A 15% concentration of surfactant was selected, based on the toxicity of POEA and its content in the actual product [8]. Tween 20 was also used at 15% according to the condition of POEA.

### 2.3. Animal Study

This study was conducted in accordance with ethical requirements and authorized by the Institutional Animal Care and Use Committee of Dankook University (approval number: DKU-22-003). Male Sprague–Dawley rats weighing 200–220 g (7 weeks old) were supplied by Raon Bio Co. (Yongsan, Republic of Korea). After an acclimatization period of seven days, the experiment was conducted. For toxicokinetic study, rats were anesthetized by intraperitoneal injection of 1.25% avertin 24 h prior to dosing of glyphosate, and a Liveo™ silicone laboratory tube (0.51 mm (I.D.) × 0.94 mm (O.D.), USA) was surgically inserted into the jugular vein for blood collecting. Fifteen rats were randomly assigned to each of the glyphosate in DW, POEA, and Tween 20 groups (*n* = 5/group). Glyphosate dissolved in DW, POEA, and Tween 20 was orally administered to rats at a dose of 50 mg/kg. After oral administration, to determine glyphosate concentrations in the blood, blood samples (200–300 μL) were collected from cannulated veins at 5, 15, and 30 min and at 1, 2, 6, 12, and 24 h, and replaced by equal volumes of heparinized saline. To obtain plasma, blood samples were centrifuged at 10,000× *g* for 10 min and the supernatant stored at −80 °C prior to analysis. After 24 h of blood collection, the rats were sacrificed by euthanizing them with CO_2_, and the liver and kidneys were removed after autopsy. The liver and kidneys were rapidly frozen in liquid nitrogen and stored at −80 °C prior to analysis.

### 2.4. Analytical Methods

For analysis of glyphosate, a Hypercarb column (100 × 2.1 mm, 5 μm) (Thermo Fisher Scientific Inc., Bremen, Germany) with Security Guard Cartridges RP-1 (4 × 3.0 mm; Phenomenex, CA, USA) was used. Chromatographic separation of the compounds was carried out using a mobile phase composed of 5 mM ammonium acetate in DW containing 1% formic acid as eluent A, and ACN containing 1% formic acid as eluent B. The flow rate and injection volumes were 0.3 mL/min and 10 μL, respectively. Initially, using 20% of B, the percentage was kept constant for 2.5 min and was allowed to increase up to 80% of B in 3.5 min. Subsequently, the later percentage was kept constant for 2 min. After reverting to the initial conditions in 1 min, the composition was kept constant for another 1 min. The detailed LC conditions for glyphosate and AMPA are summarized in Supplementary Materials Table S1.

Electrospray ionization (ESI) in positive or negative mode was used for the analytes. Multiple reaction monitoring (MRM) was performed using the negative mode, and the MRM transition was set to *m*/*z* = 168 → 62.8/78.8 for glyphosate and 110 → 62.8/78.8 for AMPA [22]. The internal standard, 4-hydroxyacetanilide was analyzed using the positive mode, and the MRM transition was set to *m*/*z* = 152.10 → 110.20 [23]. The MRM was set up with the following ionization source parameters: curtain gas (20 L/min), ion spray voltage (±4200 V), source temperature (550 °C), ion source gas 1 (50 L/min), and ion source gas 2 (55 L/min). These parameters are summarized in Table S2.

### 2.5. Calibration Standards

The study was conducted in accordance with the guidelines for the validation of bio-sampling methods of the Korea Ministry of Food and Drug Safety [24]. Since glyphosate and AMPA are soluble in water, a stock concentration of 10 mg/mL was prepared with DW. Glyphosate and AMPA were prepared at concentrations of 10, 20, 40, 100, 200, 300, and 400 µg/mL by diluting with a stock solution. The calibration solutions were prepared by mixing 15 µL of diluted solution (glyphosate, 7.5 µL + AMPA, 7.5 µL) into 135 µL of blank plasma. The final concentrations of the calibration solutions were 0.5, 1, 2, 5, 10, 15, and 20 µg/mL. Fifty microliters of calibration solution (for each concentration), 50 μL of DW, and 100 μL of IS (10 μg/mL in ACN) were placed in an e-tube and vortexed for 30 s. The mixture was then centrifuged at 13,000 rpm for 10 min. The supernatant (180 μL) was transferred to another tube and 300 μL of dichloromethane was added. It was then mixed for 30 s using a vortex mixer and centrifuged at 13,000 rpm for 10 min. Finally, 100 μL of supernatant was filtered using a 0.2 μm polytetrafluoroethylene (PTFE) filter (ADVANTEC, Dublin, CA, USA), and 10 μL of the filtered sample was injected for glyphosate analysis.

### 2.6. Accuracy and Precision at LLOQ

Accuracy is a measure of the proximity of an experimental value to the actual amount of a substance in a matrix, and precision is a measure of how close the measurements are to each other [25]. Biological samples with at least five lower limits of quantification (LLOQ) were measured and evaluated for accuracy and precision [24]. Although overall validation was not performed, the accuracy and precision were confirmed only in the LLOQ because the value fluctuated significantly at the LLOQ concentration. Accuracy was calculated based on the following formula: accuracy (%) = (measured concentration/nominal concentration) × 100. Precision was calculated as the coefficient of variation (CV) using the following formula: precision (%) = (standard deviation/mean) × 100. The accuracy should be within 80–120% of the LLOQ, and the precision should be within 20% of the LLOQ [24].

### 2.7. Sample Preparation and Analysis

The extraction method used was based on previous studies [26]. Briefly, frozen plasma samples were thawed at room temperature. Fifty microliters of plasma, 50 μL of DW, and 100 μL of IS (10 μL/mL in ACN) were placed in an e-tube and vortexed for 30 s. The mixture was then centrifuged at 13,000 rpm for 10 min. The supernatant (180 μL) was transferred to another tube and 300 μL of dichloromethane was added. It was then mixed for 30 s using a vortex mixer and centrifuged at 13,000 rpm for 10 min. Finally, 100 μL supernatant was filtered using a 0.2 μm polytetrafluoroethylene (PTFE) filter (ADVANTEC, Dublin, CA, USA), and 10 μL of the filtered sample was injected for glyphosate analysis. 

To determine the glyphosate concentration in the organs (liver and kidney), the total organ weight (liver and kidney) was measured, and three volumes of phosphate-buffered saline (PBS) were added for homogenization. The organs were then ground with a homogenizer (IKA-Werke, Staufen, Germany) and centrifuged at 2000 rpm for 3 min, and only the supernatant was prepared. The supernatant (50 μL) was mixed with 150 μL of 50% ACN (IS, 10 μg/mL). The mixtures were vortexed and centrifuged at 13,000 rpm for 10 min and then filtered using a 0.2 μm polytetrafluoroethylene (PTFE) filter (ADVANTEC, Dublin, CA, USA).

### 2.8. Toxicokinetic Parameter

The mean plasma concentration-time data for the toxicokinetic parameters were analyzed in a non-compartmental model using the WinNonlin program (version 5.0.1; Pharsight Corporation, CA, USA). The terminal elimination half-life (T_1/2_) was calculated to be 0.693/λ_z_ [27]. The maximum concentration (C_max_) and time to C_max_ (T_max_) were determined from observed plasma concentration-time data. The area under the plasma concentration-time curve (AUC_0–t_) was obtained using the trapezoidal rule [27]. The area under the concentration-time curve from 0 to infinity (AUC_0–inf_) was calculated using the following formula: AUC_inf_ = AUC_last_ + C_last_/λ_z_, where C_last_ is the last measurable concentration, and λ_z_ is the terminal elimination rate constant [27]. The volume of distribution (V_d_) was calculated as V_d_ = CL/λ_z_, where CL is the clearance [27].

### 2.9. Statistical Analysis

Parameters are presented as mean ± standard deviation (SD). Statistical analysis was performed using the Prism software version 5.01 (GraphPad, San Diego, CA, USA). Differences in toxicokinetic parameters between the groups were analyzed using ANOVA (*p* < 0.05).

## 3. Results

### 3.1. Linearity of Calibration Standards

Calibration curves were obtained at seven concentrations of 0.5, 1, 2, 5, 10, 15, and 20 μg/mL. The chromatograms of the double blank, blank, and LLOQ (0.5 μg/mL) are presented in Figure S1. The FDA guidelines for the validation of analytical procedures recommend that r^2^ be used when evaluating linear relationships [28]. The slope should show a clear correlation between the response and analyte concentration. The results should not show large deviations from linearity, implying a correlation coefficient r^2^ > 0.99 [29]. The r^2^ values for glyphosate and AMPA were 0.9995 and 0.9996, respectively (Table S3).

### 3.2. Accuracy and Precision at LLOQ

The average accuracy of glyphosate was determined to be 113.4%. The precision was determined as 7.0% based on the CV. The accuracy of AMPA was determined to be 105.2% on average. The precision was determined to be 14.9% based on CV. The presented conditions were satisfied, and the analysis method was verified (Table S3).

### 3.3. Toxicokinetics of Glyphosate

The toxicokinetics of glyphosate in DW, 15% POEA, and 15% Tween 20 were determined using a LC-MS/MS analytical method for glyphosate. In the case of glyphosate, the highest concentration of glyphosate was measured at 2 or 6 h in the DW and Tween 20 groups, and the highest concentration was measured in the POEA group at 15 or 30 min. AMPA was either below the detection limit or not detected in the plasma in all groups.

**Figure 1:**
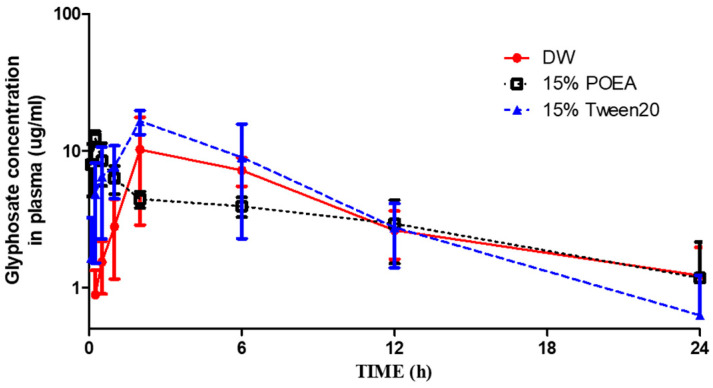
Average plasma concentration–time profiles of glyphosate in DW, 15% POEA and 15% Tween 20 groups to rats (50 mg/kg, P.O.) (*n* = 5).

The average plasma concentration-time profiles after the oral administration of glyphosate in DW, 15% POEA, and 15% Tween 20 are shown in Figure 1. The toxicokinetic parameters are summarized in Table 2. The half-life (T_1/2_) of glyphosate in the DW group was measured to be 7.42 ± 2.84 h. The highest concentration (C_max_) in plasma was 12.14 ± 5.19 μg/mL and the time to reach that concentration (T_max_) was 3.6 ± 2.2 h. The volume of distribution (V_d_) was estimated to be 4.71 ± 1.53 L/kg, and the clearance (CL) was estimated to be 0.45 ± 0.05 mL/min/kg. The area under the plasma concentration-time curve (AUC_all_) was 95.50 ± 12.62 μg∙h/mL, and AUC_inf_ was 111.31 ± 12.01 μg∙h/mL. For glyphosate in the 15% POEA group, the half-life (T_1/2_) was estimated to be 11.67 ± 4.33 h. The C_max_ was 12.78 ± 1.19 μg/mL, and the time to reach that concentration (T_max_) was 0.3 ± 0.11 h. V_d_ was 7.95 ± 0.65 L/kg and CL was estimated to be 0.60 ± 0.25 mL/min/kg. The area under the plasma curve (AUC_all_) was 75.70 ± 22.33 μg∙h/mL, and the AUC_inf_ was 101.25 ± 57.02 μg∙h/mL. Moreover, the half-life (T_1/2_) of glyphosate in 15% Tween 20 was estimated to be 7.60 ± 5.26 h. The C_max_ was 17.82 ± 2.89 μg/mL and the time to reach that concentration (T_max_) was 2.8 ± 1.78 h. V_d_ was 3.71 ± 1.66 L/kg and CL was estimated to be 0.38 ± 0.15 mL/min/kg. The area under the plasma curve (AUC_all_) was 123.26 ± 32.78 μg∙h/mL, and the AUC_inf_ was 146.04 ± 55.16 μg∙h/mL.

### 3.4. Glyphosate Concentrations in Organ

**Figure 2:**
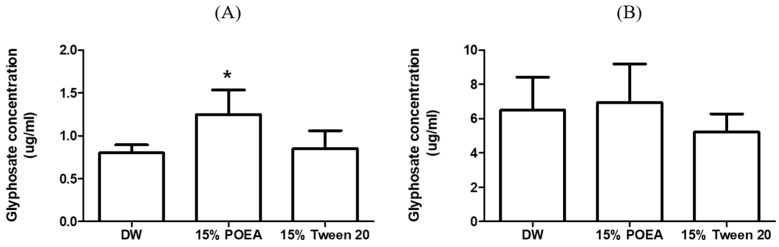
Glyphosate concentration in liver (**A**) and kidney (**B**) of DW, 15% POEA and 15% tween 20 groups for rats (*: *p* < 0.05).

For glyphosate in the DW group, the concentration in the liver was determined to be 0.8 ± 0.09 μg/mL, whereas in the 15% POEA and 15% Tween 20 groups, glyphosate concentration was found to be 1.24 ± 0.29 μg/mL and 0.85 ± 0.21 μg/mL, respectively. For glyphosate in the DW group, the concentration in the kidney was determined to be 6.5 ± 1.92 μg/mL, whereas in the 15% POEA and 15% Tween 20 groups glyphosate concentration was 6.94 ± 2.24 μg/mL and 5.23 ± 1.05 μg/mL, respectively. Glyphosate concentrations in the organs are summarized in Table 3 and Figure 2.

## 4. Discussion

Glyphosate is a broad-spectrum systemic herbicide and crop desiccant [2]. A surfactant used in a well-known glyphosate-based herbicide (Roundup**^®^**) is POEA, a nonionic surfactant. While glyphosate, the raw material for herbicides, is known to be less toxic to humans, POEA, a surfactant used in products, is known to be more toxic than glyphosate. Many studies have compared the toxicity of glyphosate raw materials and herbicides, but no studies have compared the toxicokinetics of glyphosate with other vehicles. In this study, 1% glyphosate was prepared with three vehicles, DW, POEA, and Tween 20, and the toxicokinetics of glyphosate in the three vehicles were compared by a single oral administration. To compare toxicokinetics, blood was analyzed using LC-MS/MS.

As a result of analyzing the plasma over the time intervals, there was no significant difference in all toxicokinetic parameters when the DW group and the 15% Tween 20 group were compared. Surfactants may help in faster absorption and increase the amount absorbed, but they may, in contrast, also slow it down and reduce the amount absorbed [30]. These results suggest that the surfactant Tween 20 did not affect glyphosate absorption in rats. 

However, when comparing the DW and POEA groups, notable differences were observed in T_max_ and V_d_ values, where the T_max_ was found to be 3.6 ± 2.2 h for the DW group, but 0.3 ± 0.11 h for the POEA group, which is nearly 10 times faster. This can be attributed to the fact that the POEA surfactant was involved in absorbing glyphosate more rapidly. As evidence, it was suggested that EFSA has a slight synergistic effect when considering the difference in the toxicological profile of glyphosate and combination formulations, but POEA can affect the oral absorption of glyphosate by about 20% [31]; also, the V_d_ values were about twice that of the POEA group compared to the DW group. V_d_ is the volume of the distribution value, meaning that the substance is more likely to be distributed in organs other than blood [32]. Through additional experiments, the concentration of glyphosate in the target organs (liver and kidney) was determined. There was no significant difference in the kidneys of all groups, but the POEA group showed more glyphosate residues in the liver than in the DW and Tween 20 groups (*p* < 0.05). In all groups, the concentration of glyphosate in the kidney was approximately four times greater than that in the liver, which could be seen as a target organ rather than the liver. One previous study compared the renal toxicity between Roundup**^®^** herbicide (product) and glyphosate in rats [33]. In one group, Roundup**^®^** was orally administered at glyphosate concentrations of 3.6, 50.4, and 248.4 mg/kg for 12 weeks, and in the other group, glyphosate was administered orally at 3.6, 50.4, and 248.4 mg/kg for 12 weeks. Rats exposed to Roundup**^®^** accumulated more glyphosate residues in the kidney. Serious histopathological lesions were observed only in the kidneys of this group. In this study, the presence of more glyphosate residues in the kidneys of rats exposed to Roundup**^®^** confirmed that POEA, a surfactant used in the product, was involved in the transport of glyphosate into organs. Therefore, these results suggest that the V_d_ value was larger in the POEA group.

For AMPA, a glyphosate metabolite, concentrations were below the detection limit or were not detected in any group. There are two main reasons for this result. First, the peak could not be identified because the detection limit was set high for the analysis. Glyphosate and AMPA are difficult to analyze because they are polar substances and do not contain chromophores [34]. Most of the previously-reported studies have used derivatization or solid phase extraction (SPE) columns [35]. However, the intermediate process of derivatization is cumbersome and time-consuming [34]. Therefore, in this study, an analytical method that did not go through the intermediate process of derivatization was performed, and there was a limit in obtaining a LLOQ concentration as low as that of previous studies analyzed by derivatization. Second, glyphosate is not metabolized [36]. Similar results were obtained in previous glyphosate toxicokinetic studies [36]. These results suggest that glyphosate did not induce metabolism and essentially no metabolites of glyphosate were formed due to the presence of 100% of the parent compound [37].

The predicted result before the experiment was that the presence of POEA would have a significant effect on the toxicokinetics of glyphosate and consequently lead to acute toxicity. Results showed that the absorption (T_max_) and organ distribution (V_d_) of glyphosate were affected by POEA. The toxicokinetic parameters commonly considered are the area under the concentration-time response curve (AUC) and/or C_max_ (maximum concentration). An increase in AUC and/or C_max_ requires reconsideration in terms of toxicity in animals [38]. In this study, there was no difference in C_max_ and AUC values between the DW and POEA groups individually. These results indicate that the acute toxicity of glyphosate is not significantly affected by POEA. It can be concluded that POEA toxicity itself can be attributed to the acute toxicity of glyphosate-containing products.

## 5. Conclusions

Glyphosate (1%) was prepared using DW, 15% POEA and 15% Tween 20 and administered orally once to rats. Glyphosate in POEA was absorbed about 10 times faster than DW and Tween 20, and the volume of distribution was also doubled. These results suggest that POEA, a surfactant, affects glyphosate absorption and distribution in the organs. However, no differences were observed in the C_max_ and AUC of glyphosate between the DW and POEA groups. These results indicate that the acute toxicity of glyphosate is not significantly affected by POEA. It can be concluded that POEA toxicity itself can be attributed to the acute toxicity of glyphosate-containing products. Therefore, it is recommended to replace glyphosate-containing products with safer vehicles or surfactants, such as Tween 20 rather than using highly toxic POEA surfactants in products.

## Figures and Tables

**Table 1:** Physicochemical properties for glyphosate and its metabolite, aminomethylphosphonic acid (AMPA).

	Glyphosate	AMPA
IUPAC Name ^1^	2-(Phosphonomethylamino) acetic acid	Aminomethylphosphonic acid
CAS NO. ^2^	1071-83-6	1066-51-9
Empirical formula	C_3_H_8_NO_5_P	CH_6_NO_3_P
Structural formula	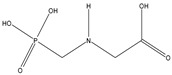	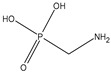
MW ^3^	169.07 g/mol	111.04 g/mol
Log Pow ^4^	−3.4	−4.7
Solubility	5 to 10 mg/mL at 64° F In water, 10.5 g/L in water at pH 1.9 and 20 °C	-
Synonyms	N-(Phosphonomethyl)glycine Roundup^®^	1-Aminomethylphosphonic acid Aminomethanephosphonic acid

^1^ International Union of Pure and Applied Chemistry; ^2^ Chemical Abstract Service Register Number; ^3^ Molecular Weight; ^4^ Octanol–water partition coefficient.

**Table 2:** Toxicokinetic parameters of glyphosate following oral administration at dose of 50 mg/kg to rats.

Parameter	DW	15% POEA	15% Tween 20
T_1/2_ (h)	7.42 ± 2.84	11.67 ± 4.33	7.60 ± 5.26
Tmax (h)	3.60 ± 2.20	0.30 ± 0.11 *	2.80 ± 1.78
C_max_ (μg/mL)	12.14 ± 5.19	12.78 ± 1.19	17.82 ± 2.89
AUC_all_ (μg∙h/mL)	95.50 ± 12.62	75.70 ± 22.33	123.26 ± 32.78
AUC_inf_ (μg∙h/mL)	111.31 ± 12.01	101.25 ± 57.02	146.04 ± 55.16
Vd (L/kg)	4.71 ± 1.53	7.95 ± 0.65 *	3.71 ± 1.66
CL (mL/min/kg)	0.45 ± 0.05	0.60 ± 0.25	0.38 ± 0.15

T_1/2_ (h), the terminal elimination half-life; T_max_ (h), the time to reach the peak plasma concentration. C_max_ (g/mL), the peak plasma concentration; AUC_all_ (μg∙h/mL), the area under the curve from zero to the last observation time point; AUC_inf_ (μg∙h/mL), the area under the curve from zero to infinity time; V_d_ (L/kg), Volume of distribution; CL (mL/min/kg), systemic clearance; * *p* < 0.05.

**Table 3:** Glyphosate concentrations (μg/mL) of liver and kidney in DW, 15% POEA and 15% Tween 20 groups in rats.

N	Liver			Kidney		
	DW	15% POEA	15% Tween 20	DW	15% POEA	15% Tween 20
1	0.92	1.68	0.52	4.60	9.48	5.24
2	0.75	1.15	1.09	8.84	7.45	5.83
3	0.83	1.37	0.92	5.32	8.50	5.96
4	0.71	1.11	0.86	7.24	4.20	5.71
5	- ^1^	0.93	0.87	- ^1^	5.08	3.42
Mean ± SD	0.8 ± 0.09	1.24 ± 0.29 *	0.85 ± 0.21	6.5 ± 1.92	6.94 ± 2.24	5.23 ± 1.05

^1^: One sample was lost; *: *p* < 0.05.

## Data Availability

Not applicable.

## Supplementary Materials

The following supporting information can be downloaded at: https://www.mdpi.com/article/10.3390/toxics11010067/s1, Table S1: Analytical LC condition of glyphosate and AMPA. Table S2: Optimized MS/MS parameters of glyphosate, AMPA and 4-hydroxyacetanilide (IS) and analytical MS/MS condition. Table S3: Accuracy, precision, and linearity at LLOQ of glyphosate and AMPA (0.5 μg/mL) (*n* = 5). Figure S1. Representative MRM chromatograms of glyphosate, AMPA, and 4-hydroxyacetanilide (internal standard, IS) obtained at LLOQ (0.5 μg/mL) in plasma (A), liver (B) and kidney (C).

## Abbreviations

ACN, Acetonitrile; AMPA, Aminomethylphosphonic acid; AUC, Area under the plasma concentration-time curve; CL, Systemic clearance; C_max_, peak plasma concentration; CV, Coefficient of variation; DW, Distilled water; LC, Liquid chromatography; LLOQ, Lower limit of quantitation; MFDS, Ministry of Food and Drug Safety; POEA, Polyoxyethylene tallow amine; PTFE, Polytetrafluoroethylene; T_1/2_, terminal elimination half-life; TK, Toxicokinetic; T_max_, time to reach the peak plasma concentration; V_d_, Volume of distribution.
