# Supplementary material for: Negligible Toxicokinetic Differences of Glyphosate by Different Vehicles in Rats
Source: Toxics. 2023 Jan 11;11(1):67. doi: 10.3390/toxics11010067 (PMC9861297; doi:10.3390/toxics11010067)
Supplement: Supplementary file 1 [file toxics-11-00067-s001.zip › toxics-2105550-supplementary.pdf]

toxics MDPI

Supplementary Materials: Toxicokinetic Properties of Glyphosate, a Vehicle-Dependent Herbicide, in Rats

Yu-Jin Kim, Kyu-Bong Kim

Table S1. Analytical LC condition of glyphosate and AMPA.

Instrument		
HPLC	Agilent Infinity 1290 LC	
Column	Hypercarb column (100 × 2.1 mm, 5 μm)	
Guard column	Security Guard Cartridges RP-1 (4 × 3.0 mm)	
LC condition		
Mobile Phase	A: 5 mM ammonium acetate in DW (1% formic acid)<br>B: ACN (1% formic acid)	
Column oven temperature (℃)	25	
Autosampler temperature (℃)	4	
Flow rate (mL/min)	0.3	
Running time (min)	10	
Injection volume (μl)	10	
Gradient condition		
Time (min)	A	B
0	80	20
2.5	80	20
6	20	80
8	20	80
9	80	20
10	80	20

Table S2. Optimized MS/MS parameters of glyphosate, AMPA and 4-hydroxyacetanilide (IS) and analytical MS/MS condition.

Compound	ESI mode	Precursor ion (m/z)	Product ion (m/z)	CE * (V)	DP ** (V)
Glyphosate	Negative	168	62.8	-30	-55
		168	78.8	-60	
AMPA	Negative	109.9	63	-24	-25
		109.9	78.8	-38	
4-hydroxyacetanilide (IS)	Positive	152.1	110.2	23	41

MS/MS Instrument	AB SCIEX API 4000
MS/MS condition	
Curtain gas (L/min)	20
Ion spray voltage (V)	- 4200 (negative) / 4200 (positive)
Ion source gas 1 (L/min)	50
Ion source gas 2 (L/min)	55
Source temperature (°C)	550

CE *: Collision energy; DP **: Declustering potential

Table S3. Accuracy, precision and linearity at LLOQ of glyphosate and AMPA (0.5 µg/mL) (n=5).

n	Concentration (µg/mL)	Glyphosate	AMPA
1		0.50	0.41
2		0.59	0.48
3	0.5	0.58	0.59
4		0.60	0.59
5		0.58	0.57
Mean ± SD		0.57 ± 0.04	0.53 ± 0.08
Accuracy (%)[1]		113.40	105.20
Precision (%)[2]		7.00	14.90
Linearity (r^2)[3]		0.9995	0.9996

Accuracy (%)[1] (measured concentration/nominal concentration) × 100; Precision (%)[2] (standard deviation / mean) × 100; Linearity (r^2)[3] correlation coefficient

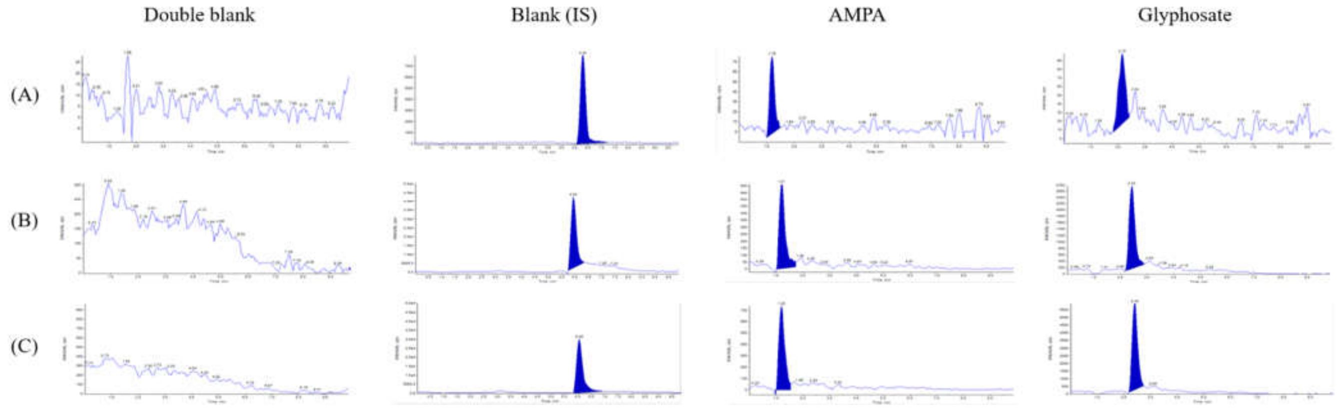

Figure S1. Representative MRM chromatograms of glyphosate, AMPA and 4-hydroxyacetanilide (internal standard, IS) obtained at LLOQ (0.5 μg/mL) in plasma (A), liver (B) and kidney (C).
